# Effectiveness of Mobile Phone-Based Interventions for Improving Health Outcomes in Patients with Chronic Heart Failure: A Systematic Review and Meta-Analysis

**DOI:** 10.3390/ijerph17051749

**Published:** 2020-03-07

**Authors:** Youn-Jung Son, Yaelim Lee, Hyeon-Ju Lee

**Affiliations:** 1 Red Cross College of Nursing, Chung-Ang University, Seoul 06974, Korea; yjson@cau.ac.kr; 2 College of Nursing, The Catholic University of Korea, Seoul 06591, Korea; ylcaregiver@gmail.com; 3 Department of Nursing, Tongmyoung University, Busan 48520, Korea

**Keywords:** heart failure, mobile phone, mortality, hospitalization, readmission, quality of life

## Abstract

Mobile phone-based interventions are increasingly used to prevent adverse health outcomes in heart failure patients. However, the effects of mobile phone-based interventions on the health outcomes of heart failure patients remain unclear. Our review aims to synthesize the randomized controlled trials (RCT) of mobile phone-based interventions for heart failure patients and identify the intervention features that are most effective. Electronic searches of RCTs published from January 2000 to July 2019 were conducted. Primary outcomes included all-cause mortality, readmission, emergency department visits, length of hospital stays, and quality of life. Secondary outcomes were self-care behaviors, including medication adherence and other clinical outcomes. A total of eight studies with varying methodological quality met the inclusion criteria and were analyzed. Voice call intervention was more frequently used compared with telemonitoring and short message services. Our meta-analysis showed that voice call interventions had significant effects on the length of hospital stays. However, no significant effects on all-cause mortality, readmission, emergency department visits, or quality of life were found. Compared to other mobile phone-based interventions, voice calls were more effective in reducing the length of hospital stay. Future studies are needed to identify which features of mobile phone-based intervention most effectively improve health outcomes.

## 1. Introduction

Heart failure (HF) is known to be a major life-threatening chronic disease with increasing prevalence, causing medical and financial problems. Worldwide, owing to advances in HF treatment and an aging population, the prevalence of HF is expected to increase by 46% by 2030 [1,2]. Despite advances in HF treatment, primary health outcomes, such as hospitalization rates and health-related quality of life (QoL), have not improved significantly [3,4]. According to recent studies, approximately 23%–58% of chronic HF patients at follow-up one year later were re-admitted to hospitals [5,6]. Chronic HF affects the elderly in particular, with 80% of HF-related hospitalizations and 90% of HF-related deaths occurring among patients aged 65 years or older [7]. HF management costs account for approximately 1%–2% of all healthcare expenditures, mostly associated with recurrent hospital admissions [1]. Therefore, the main goal of treating patients with chronic HF is to avoid adverse outcomes [8].

Chronic HF management focuses on adherence to self-care behaviors, including taking medications, symptom monitoring, and comprehensive lifestyle modification to achieve optimal health outcomes [1]. However, previous studies have reported low adherence rates in self-care behaviors, due to the complexity of self-care and lifelong requirement of medical treatment [9,10]. Thus, many studies have evaluated various types of interventions to improve HF self-care behaviors and associated outcomes [11,12,13].

In recent years, many HF studies have used mobile phones in disease management, because mobile phones are an attractive means of communication with increasingly powerful technical capabilities for providing health interventions, as well as easy access to the Internet or efficient transfer of health information [14,15]. A previous study has reported that about 96% of HF patients own mobile phones and 32% rely to some extent on a smartphone for online access and gaining health information, and report moderate self-confidence in using mobile phone applications [16]. A recent integrative review of 11 studies demonstrated that mobile health (mHealth) technology via mobile phone, such as tracking apps, offered a sense of one’s own control over chronic health conditions [14]. The potential benefits of mobile phone-based interventions include ease of use anywhere at any time, cost-effective delivery, the ability to send time-sensitive messages, and the ability to link the user with others for social support [14,17]. However, evidence regarding the effectiveness of mobile phone-based intervention for improving self-care among the chronic HF population is still lacking [17].

As far as we know, there has been only one systematic review of meta-analyses about mobile phone-based intervention and health outcomes among HF patients [18]. This previous review of nine studies, with five randomized controlled trials (RCTs) and four non-RCTs, reported that the impacts of mobile phone-based interventions on mortality, readmission, length of hospital stays, QoL, and self-care were inconsistent. However, a quantitative meta-analysis was not included in the previous review [18]. Additionally, the previous review included a telemonitoring system using a smartphone, as a feature of a wireless and mobile device. Therefore, we carried out an updated systematic review and a meta-analysis of RCTs to estimate the effects of mobile phone-based HF intervention, targeted on improving self-care, on health outcomes.

## 2. Methods

### 2.1. Search Strategies

This systematic review followed the Preferred Reporting Items for Systematic Reviews and Meta-Analyses (PRISMA) guidelines [19]. The PICOT format (P = participants, I = intervention, C = comparators, O = outcomes, T = type of study) was used to formulate the research question [20]. The PICOT research question of this review on adult patients with HF was “Does the intervention group with a mobile phone-based intervention (Intervention) among HF patients (Participants) have better health outcomes (Outcomes) than the control group given standard care (Comparison) in RCTs (Type of studies)?”

To identify all relevant articles published between January 2000 and July 2019, we conducted an extensive electronic literature search in PubMed, PsychINFO, Cochrane, CINAHL, EMBASE, Web of Science, and IEEE. The Boolean technique was used for entering keywords in each database. The following medical subject heading (MeSH) terms and keywords were used: (“heart failure” OR “cardiac failure” OR “congestive heart failure” OR “heart decompensation” OR “myocardial failure”) AND (“mobile phone” OR “smartphone” OR “cellular phone” OR “mHealth, text messaging” OR “short messaging service” OR “SMS” OR “mobile app” OR “mobile application”) AND (“intervention” OR “controlled trial” OR “RCT”). Gray literature was searched for additional potentially relevant articles. A manual search was also performed.

### 2.2. Study Selection

The inclusion criteria of this review were studies (1) published in English, (2) being original research, and (3) including adults (over 18 years old) who were receiving treatments for HF with preserved or reduced ejection fraction. Restrictions on the type of setting were not imposed. Studies excluded were (1) observational studies, reviews, conference abstracts, and letters; (2) studies with no outcome; (3) studies without a comparison group; (4) studies on acute HF; and (5) studies with insufficient information on mobile phone-based intervention, such as delivery via mobile phone and type of intervention.

**Figure 1:**
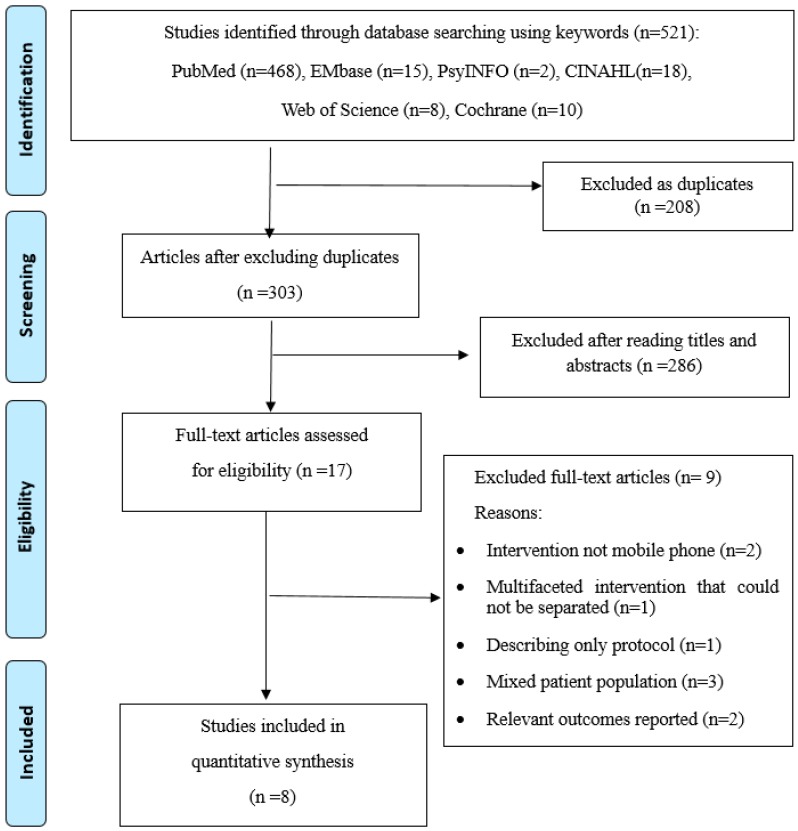
Flow chart of the systematic review of the literature selection process for the study.

Title and abstract reviews were conducted on the 303 articles, after excluding the duplicates from the initial search. A total of 286 articles were excluded for being irrelevant to the purpose of our systematic review (i.e., examining the effects of mobile phone-based HF intervention that targeted improving self-care on health outcomes). The full text of the remaining 17 articles was reviewed. A total of nine articles were excluded for the reasons described in Figure 1. To ensure reliability, two reviewers (Y.J.S. and H.J.L.) independently reviewed for inclusion. Any disagreements encountered were subsequently resolved by the third reviewer (Y.L.).

### 2.3. Data Extraction

All outcomes data were reviewed independently by two reviewers (Y.J.S. and H.J.L.) and extracted as characteristics of the included study. Each selected article was recorded with the first author’s last name, publication year, study location, participant characteristics (e.g., sample size, mean age, sex, New York Heart Association (NYHA) functional class), mobile technology (e.g., type of intervention, frequency, duration of intervention), intervention group details, control group details, outcomes, and main findings (Table 1). Primary outcomes included all-cause mortality, readmission, emergency department (ED) visits, lengths of hospital stays, and QoL [1,21,22,23]. Secondary outcomes were self-care behaviors, including medication adherence and other clinical outcomes [24,25].

### 2.4. Assessment of Methodological Quality

The risk of bias (RoB) assessment tool for RCTs was used to assess the quality of all included studies [34]. RoB is comprised of six domains—selection, performance, detection, attrition, reporting, and other bias—and was rated as high, low, or unclear risk. Included studies were independently evaluated by two reviewers (Y.J.S. and H.J.L.) for quality. Any discrepancies in quality assessment were resolved through discussion with a third reviewer (Y.L.).

### 2.5. Data Synthesis

Estimates of individual effect sizes were calculated and pooled using Comprehensive Meta-analysis software (version 3.0; Biostat, Englewood, NJ, USA). All-cause mortality, a dichotomous variable, was used to report results as risk ratios (RRs) with 95% confidence intervals (CIs). Outcomes including readmission, ED visits, length of hospital stays, and QoL were continuous variables. Thus, the mean difference was calculated using a 95% CI to investigate the effect size of the studies. A standardized mean difference (SMD) was calculated for continuous variables that measured the same outcome. The random-effects model was utilized because of the inferred heterogeneity between the studies [35]. Heterogeneity in the results was determined by using inverse variance index (I^2^) with its 95% CI and *Q* statistics (statistical significance when *p* < 0.05).

## 3. Results

### 3.1. Study Quality Appraisal

This review showed a relatively moderate overall RoB. Four studies were rated to have a low RoB [27,29,31,32]. Three out of the eight studies were rated to have a high RoB [26,28,33], and the other studies were rated to have an unclear RoB [30]. Random sequence generation was reported in five studies [27,29,31,32,33]. However, three studies did not report how the allocation sequence was generated [27,29,33]. Blinding of participants and personnel was lacking in the majority of the studies [26,27,28,32,33]. However, the blinding of patients and healthcare personnel could have been especially difficult in mobile phone-based interventions.

### 3.2. Study Settings and Patient Characteristics

As shown in Table 1, eight RCTs with a total of 2534 patients were included in this systematic review. Seven studies were conducted in the United States and Europe [26,27,28,29,30,31,32], as well as one in China [33]. Of the patients, 1331 were allocated to the intervention group and 1203 to the control group. Among them, 1444 patients were men (57%) and 1090 were women (43%). The mean age range of the participants was 52.3 to 74.6 years. Around 68% of HF patients were in NYHA functional classes III and IV.

### 3.3. Types of Mobile Phone-Based Interventions

Interventions were categorized into three sorts: voice call (*n* = 5), telemonitoring (*n* = 3), and short message service (SMS) (*n* = 1). Although the number of reviewed articles was eight, nine interventions were noted, as the study by Chen and colleagues used two interventions (voice call and SMS) [33].

#### 3.3.1. Voice Call Interventions

Five studies [26,28,30,32,33] describe the voice call intervention. The intervention period varied from 1 month to 24 months. Voice call cycles were regularly communicated with HF patients during the study period in three studies [28,30,32]; however, in two studies, the frequency varied widely from only once to frequently [26,33]. Voice call time was used to talk directly to patients in four studies [28,30,32,33], but in the other study, family members and community agencies were contacted [26]. Nurse-led voice call interventions were conducted in two studies [28,30]. The contents of the voice call mainly pertained to the pathophysiology of HF, medication adherence, diet, HF signs and symptoms, smoking cessation, and goal setting.

#### 3.3.2. Telemonitoring Interventions

Three studies provided descriptions of the telemonitoring intervention [27,29,31]. Mobile phone-based telemonitoring collected daily data on blood pressure, heart rate, body weight, dosage of HF medication, and single-lead ECGs with a range of one to six months. Two studies contacted the patient directly via the mobile phone to obtain abnormal measurements [27,29].

#### 3.3.3. SMS Intervention

Only one study used SMS to send messages to HF patients and their caregivers regarding HF knowledge and weekly reminders for taking medication and measuring weight for one month [33].

### 3.4. Study Outcomes

#### 3.4.1. Primary Outcomes Using Meta-Analysis

##### All-Cause Mortality

**Figure 2:**
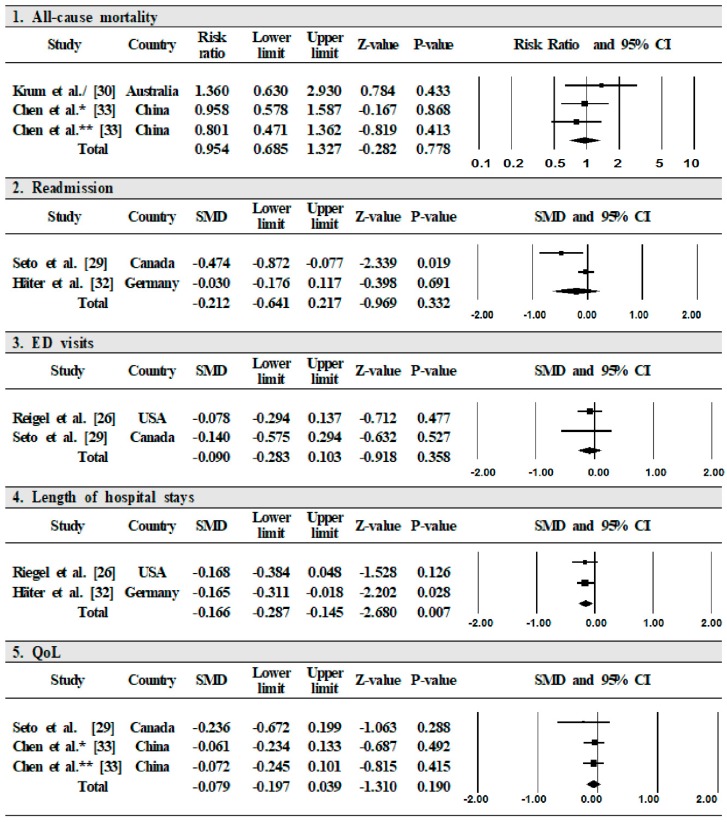
Effect of mobile phone-based interventions on health outcomes. CI = confidence interval; SMD = standard mean difference; ED visit = emergency department visit; QoL = quality of life.

Two studies examined the effects of mobile phone-based interventions on mortality [30,33]. The risk of mortality was lower in the intervention group than in the control group (RR = 0.954, 95% CI = 0.685 to 1.327). The pooled results showed low heterogeneity (*I*^2^ = 0%, *p* = 0.539) (Figure 2).

##### Readmission

In three studies, the effect of mobile phone-based interventions on readmission was reported [28,29,32]. Two studies demonstrated that the effect of the mobile phone-based intervention for HF patients was not statistically significant (SMD = −0.212, 95% CI = −0.641 to 0.217) [29,32]. The pooled results showed high heterogeneity (*I*^2^ = 76%, *p* = 0.040) (Figure 2).

##### Emergency Department Visits

Two articles measured ED visits [26,29]. Their results demonstrated that the effect of mobile phone-based interventions for HF patients was not statistically significant (SMD = −0.090, 95%CI = −0.283 to 0.103). The pooled results showed low heterogeneity (*I*^2^ = 0%, *p* = 0.802) (Figure 2).

##### Length of Hospital Stays

Two articles measured the lengths of stays in hospital [26,32]. The results demonstrated that the effect of mobile phone-based interventions for HF patients was statistically significant (SMD = −0.166, 95% CI = −0.287 to −0.145). The pooled results showed low heterogeneity (*I*^2^ = 0%, *p* = 0.978) (Figure 2).

##### Quality of Life

Two studies measured QoL using the total score of the Minnesota Living with Heart Failure Questionnaire (MLHFQ) [29,33]. The results demonstrated that the effect of mobile phone-based interventions for HF patients was not statistically significant (SMD = −0.079, 95% CI = −0.197 to 0.039). The pooled results showed low heterogeneity (*I*^2^ = 0%, *p* = 0.760) (Figure 2). Meanwhile, in Brandon et al.’s study, which conducted a repeated-measure ANOVA, mobile phone-based intervention was found to be a significant intervention over time, as demonstrated by overall QoL (F = 5.899, *p* = 0.026) [28].

#### 3.4.2. Secondary Outcomes Using Systematic Review

One study showed that voice call intervention did significantly improve self-care behaviors [28]. With regard to medication adherence, two studies showed that voice call [32] and mobile phone-based telemonitoring [31] had no significant effect on medication adherence in HF patients. Telemonitoring intervention significantly improved functional capacity, measured by the NYHA class (*p* < 0.001) [27]. On the other hand, telemonitoring had no significant effect on B-type natriuretic peptide and left ventricular ejection fraction patients [29].

## 4. Discussion

This systematic review rated the effects of mobile phone-based interventions on health outcomes in HF patients and further investigated the benefits across different mobile-based intervention modalities. Our meta-analyses showed that voice call intervention significantly shortened the length of hospital stays. However, other mobile phone-based interventions did not have significant effects on all-cause mortality, readmission, ED visits, and QoL for HF patients. The mean age of participants was over 60 years in seven of the eight reviewed articles [26,27,28,30,31,32,33], which was statistically significant in at least one of the health outcomes. This was related to the results of a systematic review on the effects of remote patient monitoring among health failure patients, in which 15 out of 19 were elderly patients [36]. Changizi and Kaveh [37] also found that mHealth interventions were effective in improving healthy behaviors in persons aged over 60, showing that the interventions were statistically significant in improving disease prevention, lifestyle changes, and cardiovascular disease management. These results support the argument that mobile phone-based interventions are feasible and can benefit the health outcomes of older HF patients.

In particular, our meta-analysis revealed that only voice call interventions, among several other features or modalities of mobile phone-based interventions, were effective in reducing the length of hospital stays, among the primary outcomes. This finding was consistent with recent reviews that tele-communications are effective in promoting adherence to cardiometabolic medications and managing long-term conditions, including clinical outcomes [38,39,40]. In addition, the direct interactions between healthcare professionals and patients through voice calls are likely to contribute to establishing confidence in providing information and receiving immediate feedback. According to a previous study [30], the interventionists, with their clinical expertise in voice call interventions for HF patients, can contribute to their participants’ adequate care and foster collaboration with healthcare teams. However, the intervention has several limitations in busy clinical settings. The healthcare providers, including the patients’ doctors or nurses, would seldom have time for making voice calls to each patient. Therefore, the voice calls are often delivered by other health-related personnel—for example, pharmacy students [41]. Interactive voice response (IVR) can be an alternative in voice call interventions. IVR is a telephone-based technology that uses touch-tone phones to enable the users to interact with the system using the telephone keypads or actual voice, through speech recognition software [42]. The human counter-speaker is replaced by a high-quality recorded interactive script with several possible answers to the patients’ inquiries [38]. Moreover, users can also leave a recorded response for more extensive feedback [43]. IVR is described as a low-cost, effective method for managing large numbers of HF patients [42]. In particular, as it does not require extensive technological skills, it may suit elderly HF patients [44].

Regarding the effect of telemonitoring and SMS, it was found that they failed to improve primary health outcomes in HF patients, as found in previous systematic reviews [18,45]. Even though patients can benefit from telemonitoring interventions or SMS interventions for monitoring values (e.g., weight, blood pressure, heart rate) or receiving reminders, it is difficult to get immediate feedback or support from healthcare professionals. A recent critical review on telemonitoring in HF patients reported telemonitoring to be ineffective in improving clinical benefits. However, it was suggested that collaboration with general practitioners may be beneficial [46]. On the other hand, Yasmin et al. highlighted that active and interactive SMS interventions would be more effective in improving patients’ health outcomes compared to passive and one-way SMS interventions [47]. Thus, telemonitoring or texting based on simple information delivery can be effective in strengthening interaction with patients and considering patients’ preferences. Furthermore, mHealth service providers need to understand the different behaviors among different user groups of mHealth services. Particularly, healthcare professionals should understand that elderly patients are likely to have limited health literacy and experience with mHealth services [48].

Our systematic review showed that the effects of voice calls, telemonitoring, or SMS interventions on mortality, readmission, ED visits, QoL, and self-care behaviors, including medication adherence and clinical indicators, were inconsistent among individual studies. With regard to self-care behaviors, one study {28} reported that nurse-led voice call intervention had a significant effect on self-care behaviors. On the other hand, two studies concerning the use of voice calls and telemonitoring reported having non-significant effects on medication adherence among HF patients [31,32]. Among self-care behaviors, long-term medication adherence can be an important component in HF disease management that directly affects hospitalization, emergency room visits, mortality, and QoL by preventing symptom aggravation [49]. These inconsistent results imply that voice call intervention can be largely determined by call frequency, length, and feedback to others [30]. Thus, healthcare providers who want to design voice call intervention should consider which characteristics of voice call are suitable to HF patients. Also, HF self-care behaviors include a complex treatment regimen, such as low salt diet, regular exercise, weight control, and symptom monitoring as well as medication adherence. Accordingly, voice calls or other types of mobile phone-based intervention may be not easy to target specific type of self-care behaviors. Moreover, such inconsistent results might be associated with the severity of HF or duration and intensity of intervention [50]. Therefore, when designing mobile phone-based interventions, it would be helpful to consider patients’ preferences, such as frequency, duration, or preferred modalities of intervention and patients’ functional status. Interestingly, combinations with more than two features of intervention were not included in this review. Future studies are needed to compare the effects between combined features and single features of mobile-based interventions. Although mobile phone-based interventions have various benefits (e.g., cost-effectiveness, effective approach, convenience), seven of eight reviewed studies were from Western countries. Since all of these were developed countries, future studies should explore effective mobile phone-based intervention programs that will benefit more HF patients through international comparisons, including those in countries with different cultures and economic statuses.

Recently published systematic reviews on mobile phone-based intervention for HF patients confirmed the qualitative synthesis of the interventions’ effects [18,36,51]. In comparison to these reviews, our study proposed objective and scientific evidence by exploring five health outcomes (mortality, readmission, QoL, hospital days, and ED visits) through meta-analysis and quantitative synthesis. In addition, our review explored diverse types of mobile phone-based interventions (voice call, telemonitoring, and SMS), unlike that by Cajita [18], where nine interventions out of 10 involved telemonitoring, even though both studies used similar search terms (i.e., Mobile Health, mHealth, Text Messages, SMS) [18]. This suggests that our review used an appropriate literature search strategy. While the above-mentioned reviews included non-RCTs, our review included only RCTs to provide the most reliable scientific evidence by minimizing any possible bias and error. Finally, our results can be utilized in future studies concerning the development of mobile phone-based interventions, as they specifically examine the types and specifics of each intervention (intervention period, frequency, the professionalism of the interventionist, and feedback) rather than simply confirming its effectiveness as a whole.

There are several limitations to this study. First, the findings lacked generalizability, due to their small sample size and small number of studies. Second, readmission and QoL were not fully meta-analyzed due to the inconsistency of statistical methods used in the reviewed past studies, and adherence and actual usage by the participants were not considered. Finally, the HF patients that were included in this study were mostly elderly, and may have found it difficult to use a mobile phone. Further studies should consider the target age of the study participants.

## 5. Conclusions

Although HF management is a major burden on health professionals in low-resource contexts, there is very little evidence demonstrating the widespread use of a mobile phone-based intervention for HF patients. Our meta-analysis demonstrated that voice call interventions can reduce the length of hospital stays of HF patients. However, based on our few trials, telemonitoring or SMS intervention did not significantly affect health outcomes. Thus, more evidence from high-quality clinical trials is required to support future mobile phone-based HF interventions. Furthermore, it is important to identify which type of mobile phone-based intervention, and what type of features (individual or combined), are more effective to improve clinical and patient-reported outcomes. Moreover, further research is needed on the tailoring of mobile phone-based interventions to the needs and sociocultural context of HF patients.

## Figures and Tables

**Table 1:** Description of studies included (*n* = 8).

Authors, (Publication Year)/Location	Participants		Contents of Mobile Technology	Intervention	Outcome Variables	Main Findings (of IG Compared to the CG)
	Intervention Group	Control Group				
Riegel et al. (2002)/United States [26]	*n* = 130	*n =* 228	·Case manager led voice calls, (median 14 voice calls for counseling, monitoring, and a supply of medications)	·IG: Nurse-led decision-support software program was used for patient education and monitoring. Additionally, printed educational material was mailed to patients monthly. -Initiation of intervention: 5 days after hospital discharge -Intervention length: 6 months ·CG: Usual care	Hospitalization, readmission, hospital days, costs, ED visits	IG scored significantly lower on hospitalization (*p* = 0.03), readmission (*p* = 0.03), hospital days (*p* = 0.01); no significant difference in ED visits.
	Mean age: 72.5	Mean age: 74.6				
	M: 62%, F: 38%	M: 46%, F: 54%				
	NYHA:	NYHA:				
	I–II = 2.4%,	I–II = 3.6%,				
	III–IV = 97.6%,	III–IV = 96.4%,				
Scherr et al. (2009)/Austria [27]	*n* = 54	*n =* 54	·Telemonitoring via mobile phone (blood pressure, heart rate, body weight, and heart failure medication on a daily basis)	·IG: Patients were asked to measure and record their BP, HR, and body weight on a daily basis, and their dosage of HF medication in their mobile phone. -Initiation of intervention: Prior to discharge. -Intervention length: 6 months. ·CG: Usual care	Survival, NYHA class, LOS	IG showed significantly high survival (*p* = 0.04), improved NYHA class (*p* < 0.001), shorter LOS (*p* = 0.04).
	Mean age: 64	Mean age: 65				
	M: 72%, F: 28%	M: 74%, F: 26%				
	NYHA:	NYHA:				
	I–II = 13%	II = 13%				
	III–IV = 87%	III–IV = 87%				
Brandon et al. (2009)/United States [28]	*n* = 10	*n =* 10	·Nurse-led voice call (5~30 min, weekly for 2 weeks, and every 2 weeks for the following 10 weeks) for patient education and support	·IG: Nurse-led telephone-enhanced disease management -Initiation of intervention: after enrollment at inpatient and outpatient settings -Intervention length: 10 weeks ·CG: Usual care	Readmission, QoL, self-care behaviors	IG had significantly greater self-care behaviors (*p* < 0.001); no significant difference in readmission, QoL.
	Mean age: 60	Mean age: 60				
	M: 30%, F: 70%	M: 60%, F: 40%				
	NYHA:	NYHA:				
	I–II = 70%,	I–II = 80%,				
	III–IV = 30%	III–IV = 20%				
Seto et al. (2012)/Canada [29]	*n* = 50	*n =* 50	·Telemonitoring via Bluetooth to a mobile phone (daily morning weight, blood pressure readings, as well as single-lead ECGs) ·Voice call for answering daily morning symptoms.	·IG: Telemonitoring system (daily morning weight and blood pressure readings and weekly single-lead ECGs). Technical support provided by telephone throughout the study. -Initiation of intervention: after enrollment at the outpatient clinic. -Intervention length: 6 months ·CG: Standard care	Readmission, mortality, QoL, self-care behaviors, BNP, LVEF, ED visits	IG had significantly greater QoL (*p* = 0.05); no significant difference in readmission, mortality, self-care behaviors, BNP, LVEF, and ED visits.
	Mean age: 55.1	Mean age: 52.3				
	M: 82%, F: 18%	M: 76%, F: 24%				
	NYHA:	NYHA:				
	I–II = 42%,	I–II = 44%				
	III–IV = 58%	III–IV = 56%				
Krum et al. (2013)/Australia [30]	*n* = 188	*n =* 217	·Voice call for answering monthly HF clinical status, medical management status, social status, and receiving advice	·IG: Patients were asked to voice call at least monthly and answer questions about the heart failure clinical status, medical management status, and social status. Additionally, patients were able at any time to dial and receive advice about management of heart failure. -Initiation of intervention: After enrollment at outpatient clinic. -Intervention length: 12 months ·CG: Individualized patient diary	All-cause mortality, hospitalization	IG had significantly less frequent hospitalization (*p* = 0.006); no significant difference in mortality.
	Mean age: 73	Mean age: 73				
	M: 62%, F: 38%	M: 64%, F: 36%				
	NYHA:	NYHA:				
	I–II = 58%,	II = 60%,				
	III–IV = 42%	III–IV = 40%				
Goldstein et al. (2014)/United States [31]	*n* = 28	*n =* 30	·Reminders (for medication taken on a daily basis) via mobile phone ·Providing information of medication via mobile phone	·IG: Medication adherence app -Initiation of intervention: Unspecified -Intervention length: 1 month ·CG: Patients were asked to use an electronic pillbox to remind them to take medication	Medication adherence	No significant difference in medication adherence.
	Mean age: 69	Mean age: 69.6				
	M: 68%, F: 32%	M: 63%, F: 37%				
	NYHA: none	NYHA: none				
Härter et al. (2016)/Germany [32]	*n* = 364	*n =* 354	·Health coach-led voice call (every 6 weeks for 24 months) for counseling, monitoring, and support	·IG: Telephone-based health coaching -Initiation of intervention: unspecified -Intervention length: 24 months ·CG: Usual care	Readmission, hospital days, medication adherence	IG showed significantly lower readmission (*p* = 0.012). No significant difference in hospital days, medication adherence.
	Mean age: 70.6	Mean age: 71.0				
	NYHA: none	NYHA: none				
Chen et al. (2019)/China [33]	Intervention 1 (SMS)		IG1: Sending text message via mobile phone (weekly for 1 month) for patient education and medication reminder (taking medicine, weighing) IG2: Voice call (once for 1 month) for patient education and counseling	IG1: Educational and reminder text message -Initiation of intervention: within 10 days after hospital discharge -Intervention length: 1 month IG2: Nurse-led voice call -Initiation of intervention: within 30 days after hospital discharge -Intervention length: 1 month ·CG: Standard care	All-cause mortality, hospitalization, QoL, self-care behaviors	No significant difference in mortality, QoL, self-care behaviors
	*n* = 252	*n* = 260				
	Mean age: 60	Mean age: 61				
	M: 58%, F: 42%	M: 57.3%, F: 42.7%				
	NYHA:	NYHA:				
	I–II = 30.6%,	I–II = 33.8%,				
	III–IV = 69.4%	III–IV = 66.2%				
	Intervention 2 (STS)					
	*n* = 255					
	Mean age: 62					
	M: 55%, F: 45%					
	NYHA:					
	I–II = 30.2%,					
	III–IV = 69.8%					

Note: IG = intervention group; CG = control group; M = male; F = female; NYHA = New York Heart Association; ED visits = emergency department visits; BP = blood pressure; HR = heart rate; HF = heart failure; LOS = length of stay; QoL = quality of life; ECG = electrocardiogram; BNP = B-type natriuretic peptide; LVEF = left ventricular ejection fraction; app = application; SMS = short message service; STS = structured telephone support.
